# Supplementary material for: Identification of Freezing-Responsive microRNAs and Their Targets in Chinese Jujube by Small RNA and Degradome Sequencing
Source: Int J Mol Sci. 2026 Sep 4;27(17):7912. doi: 10.3390/ijms27177912 (PMC13566890; doi:10.3390/ijms27177912)
Supplement: Supplementary file 1 [file ijms-27-07912-s001.zip › Supplementary Material S2/novel_miR_128.pdf]

Provisional ID	:	novel_miR_128
Score total	:	0.4
Score for star read(s)	:	-1.3
Score for read counts	:	0
Score for mfe	:	0.1
Score for randfold	:	1.6
Score for cons. seed	:	
Total read count	:	92
Mature read count	:	92
Loop read count	:	0
Star read count	:	0

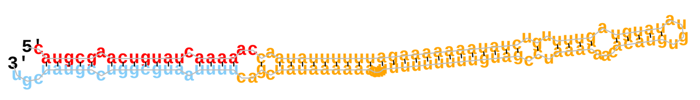

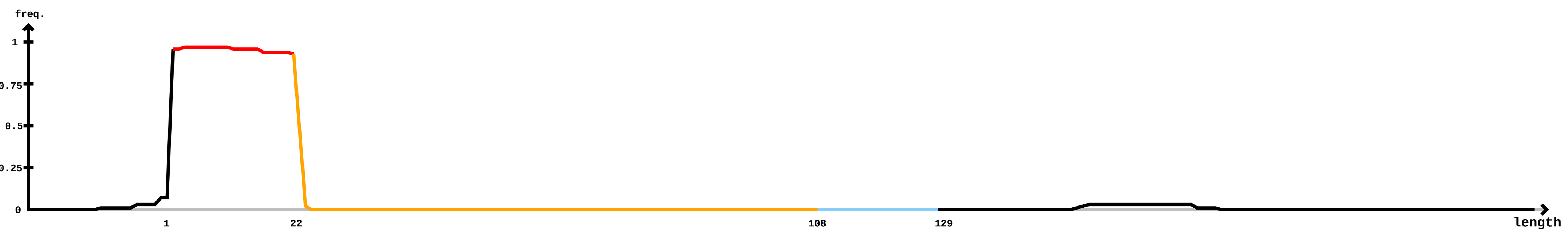

Mature Star

```
5'- uggauauuuuacggaucauccacaugcgaacuguaucaaaaaccaauauuuuuuagaaaaaaauaucuguuuugauguauauguguacacaacaaauccgauguuuuuuuuuuucaaaaaauaucgacuuuuaaugcgguccguaucgu aaaacuauuuuauauauaaauauauauauauauaugucgagucauguugacaauuuuuauauucggauuuaaaauucaaaaaagauuuaaagauagauaa -3'
    ......(((((((.........(((((.(((((((.((((..(.((((((((((((((((((((((.(.((((.(((((....)))))...)))).).))))))))))))....))))))))).)..)))).))))))).))))))))))))(((((((((((((......)))))))))..((((((......))))))...........(((((...)))))............))))))....
```

exp

read	reads	mm	sample
acggaucauccacaugcgaacu	1	0	LT-1
cauccacaugcgaacuguauc	2	0	LT-2
cacaugcgaacuguaucaaaaac	1	0	LT-1
cacaugcgaacuguaucaaaaac	1	0	LT-3
cacaugcgaacuguaucaaaaac	1	0	JCK-2
cacaugcgaacuguaucaaaaac	1	0	JCK-3
caugcgaacuguaucaaaaa	1	0	LT-3
caugcgaacuguaucaaaaac	1	0	JCK-3
caugcgaacuguaucaaaCac	1	1	LT-2
caugcgaacuguaucaaaaac	5	0	LT-1
caugcgaacuguaucaaaaac	6	0	JT-2
caugcgaacuguaucaaaaac	12	0	LT-2
caugcgaacuguaucaaaaac	6	0	JCK-2
caugcgaacuguaucaaaaac	2	0	JT-3
caugcgaacuguaucaaaaac	10	0	LT-3
caugcgaacuguaucaaaaaA	1	1	JT-2
caugcgaacuguaucaaaaac	32	0	LCK-3
caugcgaacuguaucaaaaacc	2	0	LT-2
caugcgaacuguaucaaaaCcc	1	1	LT-2
caugcgaacuguaucaaaaacc	2	0	JCK-2
caugcgaacuguaucaaaaacU	2	1	LT-3
caugcgaacuguaucaaaaacc	1	0	JCK-3
caugcgaacuguaucaaaaacca	2	0	LT-3
ugcgaacuguaucaaaaac	1	0	LT-1
uauauauauauaugucgagucaug	1	0	JCK-2
auauauauauauguAgagu	1	1	LT-3
uauauauauauguAgagu	1	1	LT-3
